# Analysis of *Nkx3.1*:Cre-driven *Erk5* deletion reveals a profound spinal deformity which is linked to increased osteoclast activity

**DOI:** 10.1038/s41598-017-13346-8

**Published:** 2017-10-16

**Authors:** Carolyn J. Loveridge, Rob J. van ’t Hof, Gemma Charlesworth, Ayala King, Ee Hong Tan, Lorraine Rose, Anna Daroszewska, Amanda Prior, Imran Ahmad, Michelle Welsh, Ernest J. Mui, Catriona Ford, Mark Salji, Owen Sansom, Karen Blyth, Hing Y. Leung

**Affiliations:** 1 Institute of Cancer Sciences, College of Medical, Veterinary and Life Sciences, University of Glasgow, Bearsden, Glasgow G61 1BD UK; 2 Beatson Institute for Cancer Research, Bearsden, Glasgow G61 1BD UK; 3 Institute of Ageing and Chronic Disease, University of Liverpool, WH Duncan Building, West Derby Street, Liverpool, L7 8TX UK; 4 Centre for Molecular Medicine, MRC IGMM, University of Edinburgh, Edinburgh, EH4 2XU UK; 5 College of Medical, Veterinary and Life Sciences, University of Glasgow, Glasgow, G61 1QH UK

## Abstract

Extracellular signal-regulated protein kinase 5 (ERK5) has been implicated during development and carcinogenesis. *Nkx3.1*-mediated Cre expression is a useful strategy to genetically manipulate the mouse prostate. While grossly normal at birth, we observed an unexpected phenotype of spinal protrusion in *Nkx3.1*:Cre;*Erk5* ^fl/fl^ (*Erk5* ^fl/fl^) mice by ~6–8 weeks of age. X-ray, histological and micro CT (µCT) analyses showed that 100% of male and female *Erk5* ^fl/fl^ mice had a severely deformed curved thoracic spine, with an associated loss of trabecular bone volume. Although sex-specific differences were observed, histomorphometry measurements revealed that both bone resorption and bone formation parameters were increased in male *Erk5* ^fl/fl^ mice compared to wild type (WT) littermates. Osteopenia occurs where the rate of bone resorption exceeds that of bone formation, so we investigated the role of the osteoclast compartment. We found that treatment of RANKL-stimulated primary bone marrow-derived macrophage (BMDM) cultures with small molecule ERK5 pathway inhibitors increased osteoclast numbers. Furthermore, osteoclast numbers and expression of osteoclast marker genes were increased in parallel with reduced *Erk5* expression in cultures generated from *Erk5* ^fl/fl^ mice compared to WT mice. Collectively, these results reveal a novel role for *Erk5* during bone maturation and homeostasis *in vivo*.

## Introduction

ERK5 (MAPK7 or BMK) belongs to the family of mitogen-activated protein kinases (MAPKs), members of which typically function as signalling nodes to integrate information from extracellular stimuli and different intracellular signalling cascades. Through this activity, MAPKs control numerous biological processes during development and homeostasis, including cellular proliferation, differentiation and survival. ERK5 has a uniquely large C-terminal domain, which contains nuclear localisation and export signals required for dynamic shuttling of ERK5. On the other hand, the N-terminal catalytic domain of ERK5 shares a 50% homology with ERK1/2 and inhibitors developed against MEK1/2, such as PD98059 and U0126, have been demonstrated to have off-target effects on the MEK5/ERK5 pathway. Unlike other members of the MAPK family, MEK5 is the only MEK (MAPK kinase) reported to directly interact with and activate ERK5 via phosphorylation^1–3^.

Deletion of *Mek5* and *Erk5* in mice has revealed their essential role during development, where almost identical phenotypes are observed^4–7^. *Erk5* null mice die around embryonic day 10.5 due to cardiovascular defects with impaired vasculogenesis and angiogenesis. This is consistent with findings that ERK5 plays a critical role in endothelial cell function^8,9^. Growth in the head region in these mice was severely retarded. In addition, ERK5 has been implicated in the physiology of neurones^10^, muscle^11^ and immune cells^12–14^ by controlling proliferation, differentiation and cell survival. ERK1/2 and ERK5 have distinct roles in the regulation of brain-derived neurotrophic factor expression^15^. In addition to its essential role in development, there is an increasing body of evidence that ERK5 plays a role in tumour development (reviewed in ref.^16^) and previous work from our laboratory has implicated ERK5 as a key driver for prostate carcinogenesis^17–19^. Hence, we initiated experiments to test the impact of *in vivo Nkx3.1*:Cre-mediated *Erk5* deletion in a transgenic mouse model^20,21^.

Formation of the axial skeleton begins with the subdivision of paraxial mesoderm into somites. Signals (*sonic hedgehog*) emanating from the notochord influence the early somites to form the sclerotome. Sclerotomal cells form the beginnings of the vertebrae. Vertebral structures subsequently develop and segment under the influence of various cellular signals, including *Pax-9* and homeobox-containing genes^22–24^. A previous study to examine the pattern of *Nkx3.1* mediated Cre recombinase expression, via the use of ROSA26 reporter mice, documented expression of *Nkx3.1*:Cre in a range of tissues, including somites, dorsal aspects of the spinal ridge, ribs and skull^25^. However, despite expression being noted in somites, no significant skeletal phenotype has been described in *Nkx3.1* deleted mice to date (reviewed in ref.^26^).

In this study, our novel finding is that *Nkx3.1*:Cre driven *Erk5* deletion associates with a phenotype of a severely curved spine and substantially reduced bone mass/osteopenia. We also report increased osteoclast differentiation in primary bone marrow cultures generated from wild type (WT) mice treated with small molecule ERK5 pathway inhibitors and in mice with *Nkx3.1*:Cre-mediated *Erk5* deletion compared to WT, indicating a link between *Erk5* and osteoclast activity *in vivo*.

## Results

### *Nkx3.1*-Cre *Erk5* null mice developed severe spinal abnormalities

**Figure 1:**
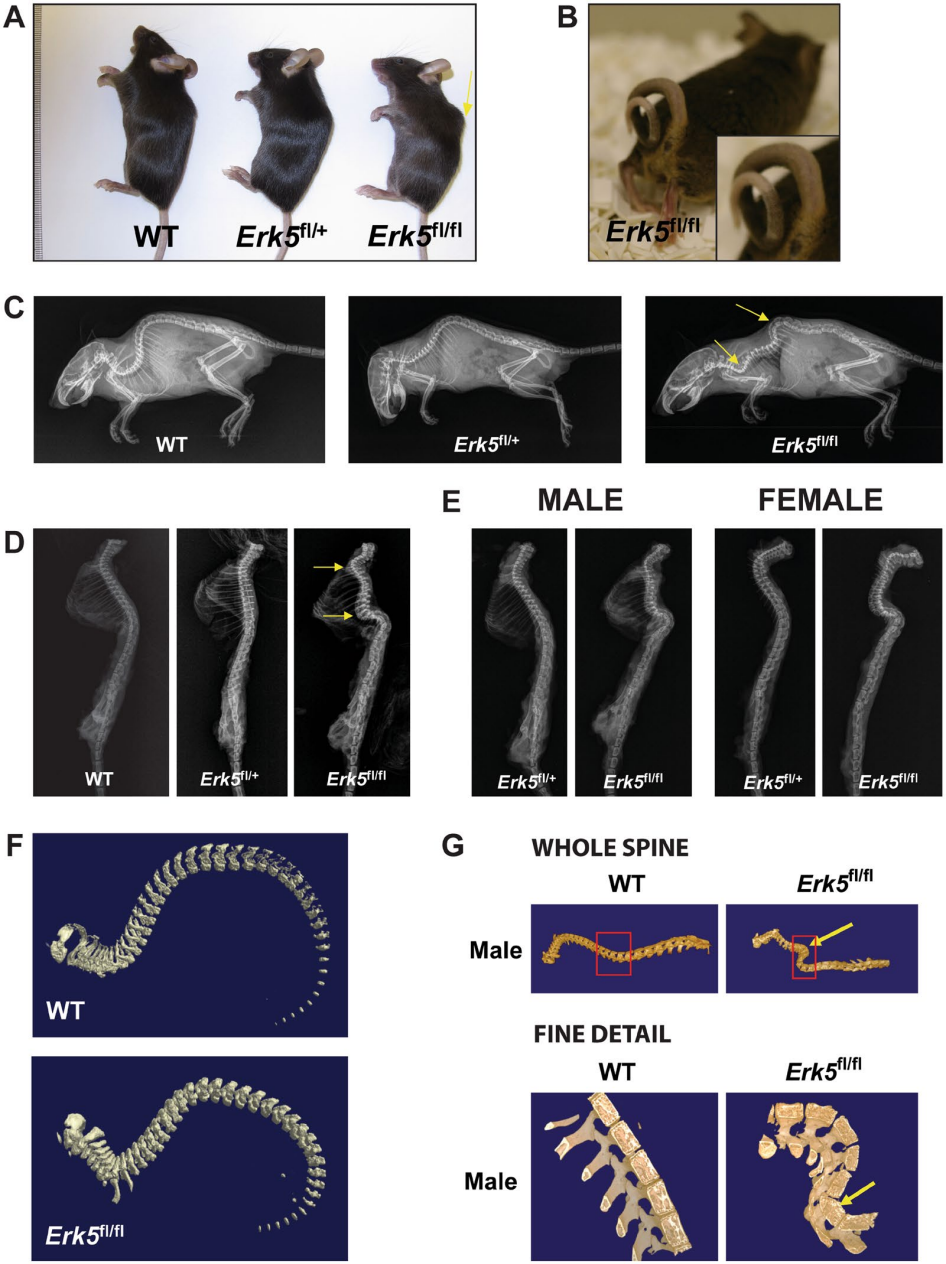
*Erk5* ^fl/fl^ mice exhibit spinal protrusion, curled tail and have severe spinal deformities in the thoracic region. (**A**) Compared to WT and *Erk5* ^fl/+^ littermates, a protruding spine in *Erk5* ^fl/fl^ mice was evident in *Erk5* ^fl/fl^ mice (yellow arrow). (**B**) Curled tail was apparent in a proportion of male and female *Erk5* ^fl/fl^ mice. The image shown is of a 4.5 month old male. (**C**) Representative images of whole body X-rays of 11 week old female mice. Severely curved spine is evident in *Erk5* ^fl/fl^ mouse compared to WT and *Erk5* ^fl/fl^ mice, where no spinal deformity was seen. Yellow arrows highlight the main areas of deformity in the thoracic region of the spine. (**D**) Representative images showing longitudinal view of X-rays of isolated spines from 10–12 week old male mice. No spinal deformity was seen in WT or *Erk5* ^fl/+^ mice. As was observed in *Erk5* ^fl/fl^ female mice, a severely curved spine was apparent in *Erk5* ^fl/fl^ male mice. Yellow arrows highlight the main areas of the deformity in the thoracic region of the spine. (**E**) Representative images showing longitudinal view of X-rays of isolated spines from 7 week old male and female *Erk5* ^fl/+^ and *Erk5* ^fl/fl^ mice. Similar to 10–12 week old mice, severely curved spines were apparent in male and female *Erk5* ^fl/fl^ mice at 7 weeks of age. (**F**) Representative images of low resolution µCT of newborn pups. No spinal defect was found in *Erk5* ^fl/fl^ mice at this stage of development. (**G**) Representative images of low resolution µCT of isolated spines from male *Erk5* ^fl/fl^ mice. In the bottom panel, fine detail images of male spines are shown. Yellow arrows denote deformation of the mutant spinal column. Note the wedge-shaped deformation of the mutant vertebral body in the fine detail view shown in the lower panel. Red boxes in the top panel indicate the regions of the spines shown in finer detail in the bottom panel.

To circumvent *Erk5* null induced embryonic lethality, conditional deletion of *Erk5* was intended to target the prostate by Cre recombinase inserted into the *Nkx3.1* locus. However, we observed marked phenotypic abnormalities in male and female *Nkx3.1*:Cre;*Erk5* ^fl/fl^ mice (homozygous for the *Erk5* conditional allele), referred to hereafter as *Erk5* ^fl/fl^ mice. Newborn pups all had the same normal gross morphology, regardless of genotype, but by 10–12 weeks of age, *Erk5* ^fl/fl^ male and female mice were noticeably and significantly smaller in size compared to WT [*Nkx3.1*:Cre;*Erk5* ^+/+^ or mice which did not express Cre recombinase] and *Erk5* ^fl/+^ [heterozygous for the *Erk5* conditional allele; *Nkx3.1*:Cre;*Erk5* ^fl/+^] littermate controls (Fig. 1A and S1A and Tables S1 and S2). *Erk5* ^fl/fl^ male and female mice, from ~6–8 weeks onwards, exhibited a protruding spine when compared to WT and *Erk5* ^fl/+^ littermate controls (Fig. 1A), indicative of a potential underlying bone defect causing abnormal curvature of the thoracic spine. Overall, among 33 *Erk5* ^fl/fl^ mice (17 male and 16 female), all developed a spinal phenotype. In contrast, 17 (7 male and 10 female) *Erk5* ^fl/+^ and 42 (18 male and 24 female) WT littermate control mice did not demonstrate any clinical evidence of spinal defect. Moreover, a curled tail phenotype (Fig. 1B) was observed in ~30% *Erk5* ^fl/fl^ mice (5/17 males and 5/16 females; > 6 weeks old). There was no evidence of abnormalities in other major organs examined, including the prostates of *Erk5* ^fl/fl^ male mice (Fig. S1B).

X-ray analysis of whole body and isolated spines from 7 and 10–12 week old WT, *Erk5* ^fl/+^ and *Erk5* ^fl/fl^ male and female mice showed no major differences between *Erk5* ^fl/+^ and WT mice (Fig. 1C and D). In contrast, we observed severe spinal curvature in the thoracic region of male and female *Erk5* ^fl/fl^ mice at 7 weeks (Fig. 1E) and 10–12 weeks of age (Fig. 1C and D), regardless of curled tail phenotype. Micro-computed tomography (μCT) analysis of newborn pups (2 days after birth) demonstrated that there was no spinal defect present in *Erk5* ^fl/fl^ mice compared to WT littermates (Fig. 1F), suggesting that the defect manifests during post-embryonic skeletal maturation. μCT analysis of isolated spines from older (10–12 weeks) male (Fig. 1G) and female (Fig. S1D) *Erk5* ^fl/fl^ mice confirmed the marked curvature in the spine as seen in plain X-ray imaging. The affected vertebrae in male and female *Erk5* ^fl/fl^ mice showed striking wedge-shaped deformities, indicative of vertebral collapse, as can be seen from the detailed images of WT and *Erk5* ^fl/fl^ spines in the bottom panels of Fig. 1G and S1D. No spinal phenotype was observed in *Nkx3.1* ^*Cre/Cre*^;*Erk5* ^+/+^ mice, which were homozygous for the *Nkx3.1*:Cre allele (Fig. S1C). Collectively, these data suggest that the loss of *Erk5*, rather than *Nkx3.1*-driven Cre expression, is the causative event for the spinal phenotype.

### Trabecular bone loss in *Erk5*^fl/fl^ mice

**Figure 2:**
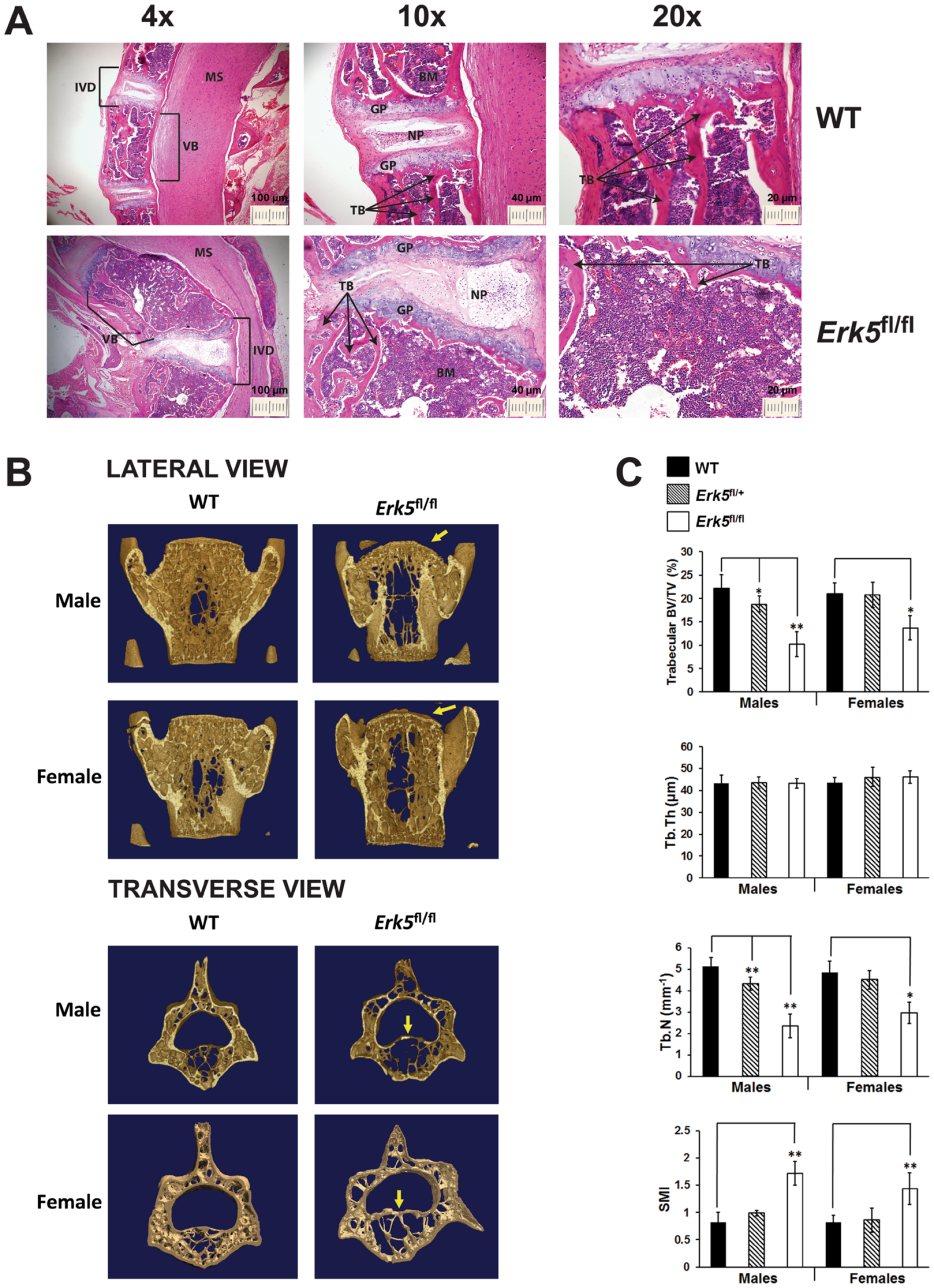
*Erk5* ^fl/fl^ mice lack trabecular bone structure and have reduced bone volume. (**A**) Representative micrographs (4 × 10x and 20x) of H + E staining of longitudinal sections from vertebrae of 5 month old female WT and *Erk5* ^fl/fl^ mice. Black arrow indicates trabecular structure, which is clearly reduced in *Erk5* ^fl/fl^ mice compared to WT. Abbreviations: BM = bone marrow; GP = growth plate; IVD = inter-vertebral disc; MS = medulla spinalis; NP = nucleus pulposus; TB = trabecular bone; VB = vertebrae. (**B**) Representative images of high resolution µCT analysis of L5 vertebrae from 11 week old male and female WT and *Erk5* ^fl/fl^ mice. Shown in top panel are lateral views; shown in bottom panel are transverse views. Yellow arrows denote some deformation of the end plates (top panel) and narrowing of the central canal in virtually cut (bottom panel) mutant vertebrae. (**C**) Quantitative analysis of high resolution µCT parameters indicate a significant reduction in trabecular BV/TV (%) and Tb.N (mm^−1^) in male and female *Erk5* ^fl/fl^ mice compared to WT and a significant increase in SMI in male and female *Erk5* ^fl/fl^ mice compared to WT. Abbreviations: BV/TV: bone volume per tissue volume; Tb.Th: trabecular thickness; Tb.SP: trabecular separation; Tb.N: trabecular number; SMI: structure model index. Shown in the graphs are means; error bars represent SEM; t test (unpaired, 2 tailed) was used to calculate p values and those with significance are shown. *p < 0.05; **p < 0.01 from same sex wild type.

Histological analysis of the thoracic spine confirmed the deformation of the thoracic vertebrae in *Erk5* ^fl/fl^ mice compared to WT mice (compare 4x images in Fig. 2A). Moreover, the vertebrae in the affected region from *Erk5* ^fl/fl^ mice were variably larger in size and structurally dis-organised when compared to WT control mice. Vertebrae from *Erk5* ^fl/fl^ mice were characterized by a significant increase in marrow space in association with a profound loss of trabecular bone mass (compare 10x and 20x images in Fig. 2A). The spinal cords showed no gross pathology associated with compression but inter-vertebral discs were enlarged with some structural malformation of nucleus pulposus (NP) tissue between collapsed thoracic vertebrae in *Erk5* ^fl/fl^ mice compared to WT mice. However, it is noteworthy that the inter-vertebral disc NP tissue located between thoracic vertebrae that had not collapsed in *Erk5* ^fl/fl^ mice appeared similar to WT (Fig. S1E).

The severe deformation of the thoracic vertebrae would make a quantitative analysis of the bone architecture difficult to interpret. Therefore μCT analysis of bone architecture was performed using the 5th lumbar vertebrae (L5) because generally, these were not badly deformed. High-resolution μCT analysis of L5 isolated from WT, *Erk5* ^fl/+^ and *Erk5* ^fl/fl^ mice (Fig. 2B) demonstrated a dramatic loss of bone volume in *Erk5* ^fl/fl^ male and female mice when compared to WT. Although the lumbar vertebrae showed no major deformation overall, some minor changes to the end plates and the central canal were observed (highlighted by yellow arrows). Both male and female *Erk5* ^fl/fl^ mice showed substantial loss of trabecular bone volume (Fig. 2B and C and Table S3). The bone loss was more pronounced in male *Erk5* ^fl/fl^ mice, where a 54% decrease in bone volume per tissue volume (BV/TV) was seen compared to WT. Female *Erk5* ^fl/fl^ mice showed a lower, but still substantial 35% decrease in BV/TV compared to WT. The loss of trabecular bone volume was accompanied by a significant reduction in trabecular number (Tb.N) in both male (54.1% compared to WT) and female (39% compared to WT) *Erk5* ^fl/fl^ mice. There was no significant difference in trabecular thickness (Tb.Th) in male and female *Erk5* ^fl/fl^ mice compared to WT. However, a deterioration of bone architecture was observed, as indicated by decreased trabecular connectivity and a significant increase in the structure model index (SMI) in both male and female *Erk5* ^fl/fl^ mice compared to WT. *Erk5* ^fl/+^ male mice also displayed reduced bone volume (15%) and trabecular number (15.8%) compared to WT, but this was less severe than *Erk5* ^fl/fl^, indicating the possibility of a dosage-dependent effect of *Erk5* loss.

To address whether bone abnormalities were present in the appendicular skeleton in addition to the axial skeleton, we analysed distal femurs by μCT (Fig. S2A and B and Table S4). There was a substantial decrease in BV/TV in both *Erk5* ^fl/fl^ and *Erk5* ^fl/+^ males (44% and 24% respectively) compared to WT. Female *Erk5* ^fl/fl^ mice also demonstrated significantly reduced BV/TV (23%) compared to WT, although this was not as marked as in the *Erk5* ^fl/fl^ male mice. *Erk5* ^fl/+^ females showed no significant bone loss in the distal femur. In keeping with trends seen for the spine, changes in bone architecture were observed in the distal femurs of male and female *Erk5* ^fl/fl^ mice. Specifically, there was a significant reduction in Tb.Th and a significant increase in trabecular pattern factor (Tb.Pf) in both male and female *Erk5* ^fl/fl^ mice compared to WT. In both *Erk5* ^fl/+^ and *Erk5* ^fl/fl^ males, there was a significant reduction in Tb.N and increase in trabecular separation (Tb.Sp) compared to WT but these parameters were not changed in female *Erk5* ^fl/+^ or *Erk5* ^fl/fl^ mice. It is noteworthy that the overall shape and morphology of the long bones of *Erk5* ^fl/fl^ mice appeared similar to WT and the growth plate in distal femurs of male *Erk5* ^fl/fl^ mice had no significant abnormalities (Fig. S2C), indicating that there was no major defect with endochondral ossification.

### Analysis of bone turnover in *Erk5*^fl/fl^ mice

**Figure 3:**
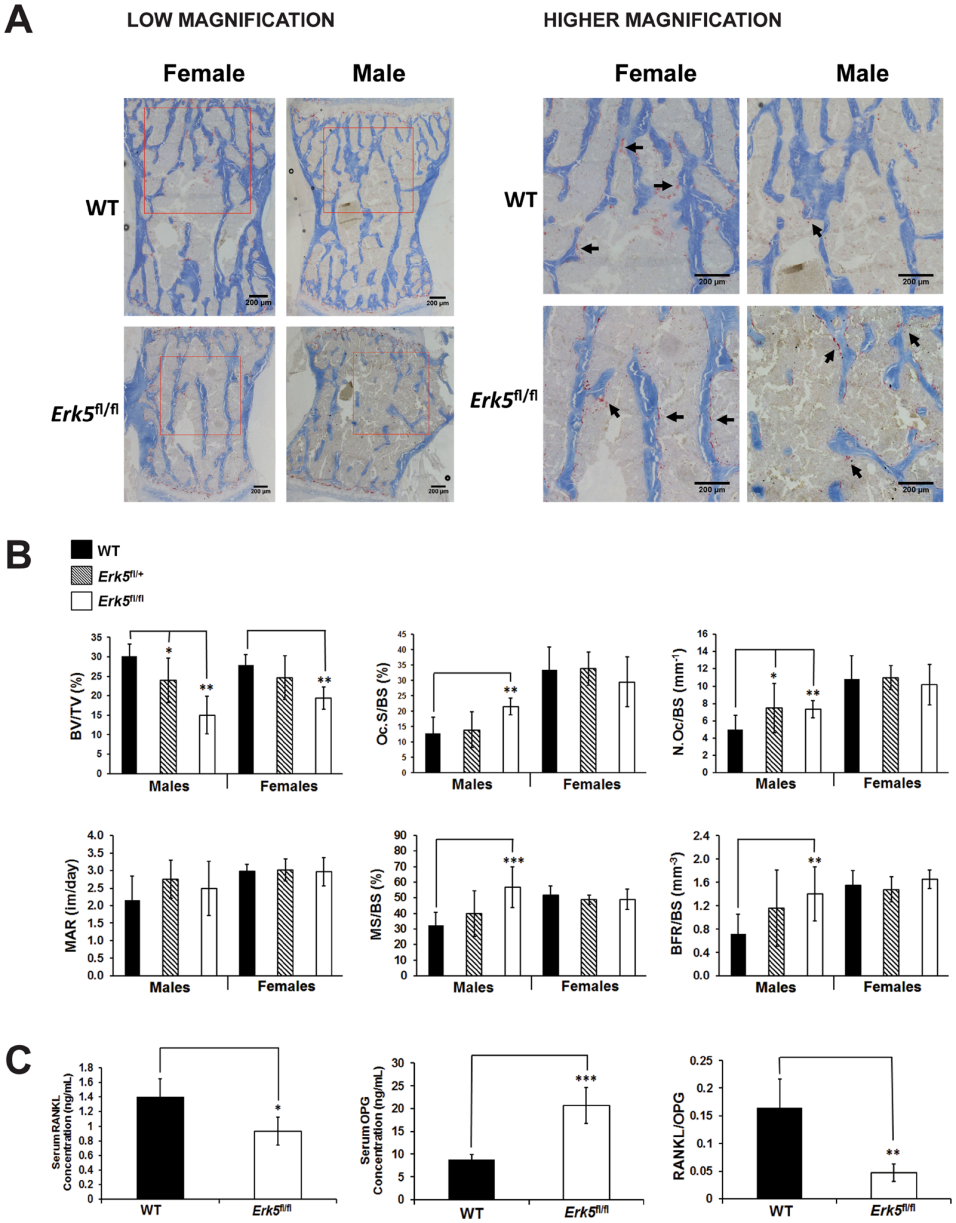
Bone histomorphometry in L5 vertebrae demonstrates increased osteoclast activity and increased bone formation parameters in male *Erk5* ^fl/fl^ mice. (**A**) Representative micrographs of TRAP staining in L5 vertebrae of 11 week old male and female WT and *Erk5* ^fl/fl^. Black arrows indicate TRAP-stained osteoclasts (stained in red). Bone tissue was counterstained with Aniline Blue. Red boxes in low magnification images in the left panel indicate the regions shown at higher magnification in the right panel. (**B**) Quantitative analysis of bone histomorphometry parameters, as assessed from TRAP staining and calcein double labelling experiments, demonstrate significantly reduced BV/TV (%) in both male and female *Erk5* ^fl/fl^ mice compared to WT. In male *Erk5* ^fl/fl^ mice, Oc.S/BS (%), N.Oc/BS (mm^−1^) and the bone formation parameters, MS/BS (%) and BFR/BS were significantly increased compared to WT littermates. There were no significant differences found in bone resorption or formation parameters in female mice. Abbreviations: BV/TV: bone volume per tissue volume; Oc.S/BS: osteoclast surface per bone surface; N.Oc/BS: number of osteoclasts per bone surface; MAR: mineral acquisition rate; MS/BS: mineralising surface per bone surface; BFR/BS: bone formation rate per bone surface. Shown in the graphs are means; error bars represent SEM; t test (unpaired, 2 tailed) was used to calculate p values and those with significance are shown. *p < 0.05; **p < 0.01; ***p < 0.001 from same sex wild type. (**C**) Ratio of RANKL/OPG is significantly reduced in *Erk5* ^fl/fl^ mice compared to WT. Serum RANKL and OPG levels were measured in 7–11 week old male WT (n = 4) and *Erk5* ^fl/fl^ mice (n = 3) by ELISA [expressed in nanograms per millilitre (ng/mL)] and the ratio of RANKL/OPG was subsequently calculated. Shown in the graphs are means; error bars represent SEM; t test (unpaired, 2 tailed) was used to calculate p values and those with significance are shown. *p < 0.05; **p < 0.01; ***p < 0.001 from same sex WT.

Bone homeostasis is achieved via the controlled activities of two major cell types: osteoblasts synthesize and deposit new bone matrix while osteoclasts break down and resorb bone. The bone loss observed in the *Erk5* ^fl/fl^ mice could therefore be due to a decrease in bone formation, an increase in bone resorption or a combination of both. In order to address this question, we performed dynamic histomorphometry on L5 vertebrae of 10–12 week old mice. In *Erk5* ^fl/fl^ males, the histomorphometry showed a significant 69% increase in osteoclast surface (Oc.S) and a 68% increase in osteoclast number (N.Oc) per bone surface (BS) (N.Oc/BS) (Fig. 3B) as assessed by tartrate-resistant acid phosphatase (TRAP) staining (Fig. 3A). There was a non-significant trend for increased osteoclast surface and a significant increase in osteoclast number in *Erk5* ^fl/+^ males, again indicating the possibility of a dosage-dependent effect of *Erk5* loss (Fig. 3B and Table S5). There was no statistically significant increase in bone resorption parameters in *Erk5* ^fl/+^ or *Erk5* ^fl/fl^ female mice. Calcein double labels were used to assess bone formation parameters. While there was no difference in mineral apposition rate, we observed a significant increase in mineralizing surface per bone surface (MS/BS) and bone formation rate per bone surface (BFR/BS) in *Erk5* ^fl/fl^ males compared to WT. There was no significant difference in any bone formation parameters in *Erk5* ^fl/+^ males or *Erk5* ^fl/+^ or *Erk5* ^fl/fl^ female mice compared to WT controls (Table S5).

Collectively, data from histomorphometry analysis are consistent with the observed bone loss in the vertebrae of *Erk5* ^fl/fl^ mice being associated with increased osteoclast activity. Key mediators of osteoclastogenesis include the receptor activator of NF-κB ligand (RANKL) and its negative regulator, osteoprotegerin (OPG). We measured the serum levels of these secreted factors in WT and *Erk5* ^fl/fl^ male mice (7–11 weeks of age). There was a significant decrease in serum RANKL levels and a significant increase in serum OPG levels, resulting in a significant decrease in the ratio of RANKL to OPG from 0.164 in WT to 0.047 in *Erk5* ^fl/fl^ male mice (p = 0.007) (Fig. 3C). It is possible that the loss of bone mass in *Erk5* ^fl/fl^ mice may have resulted in the activation of compensatory mechanisms, such as the increased production of OPG and decreased production of RANKL.

### Reduction of *Erk5* expression increased osteoclast numbers and expression of *Rank*, *Cathepsin K* (*Ctsk*) and *Nuclear factor of activated T-cells* (*Nfatc1*) in osteoclasts derived from *Erk5*^fl/fl^ mice

**Figure 4:**
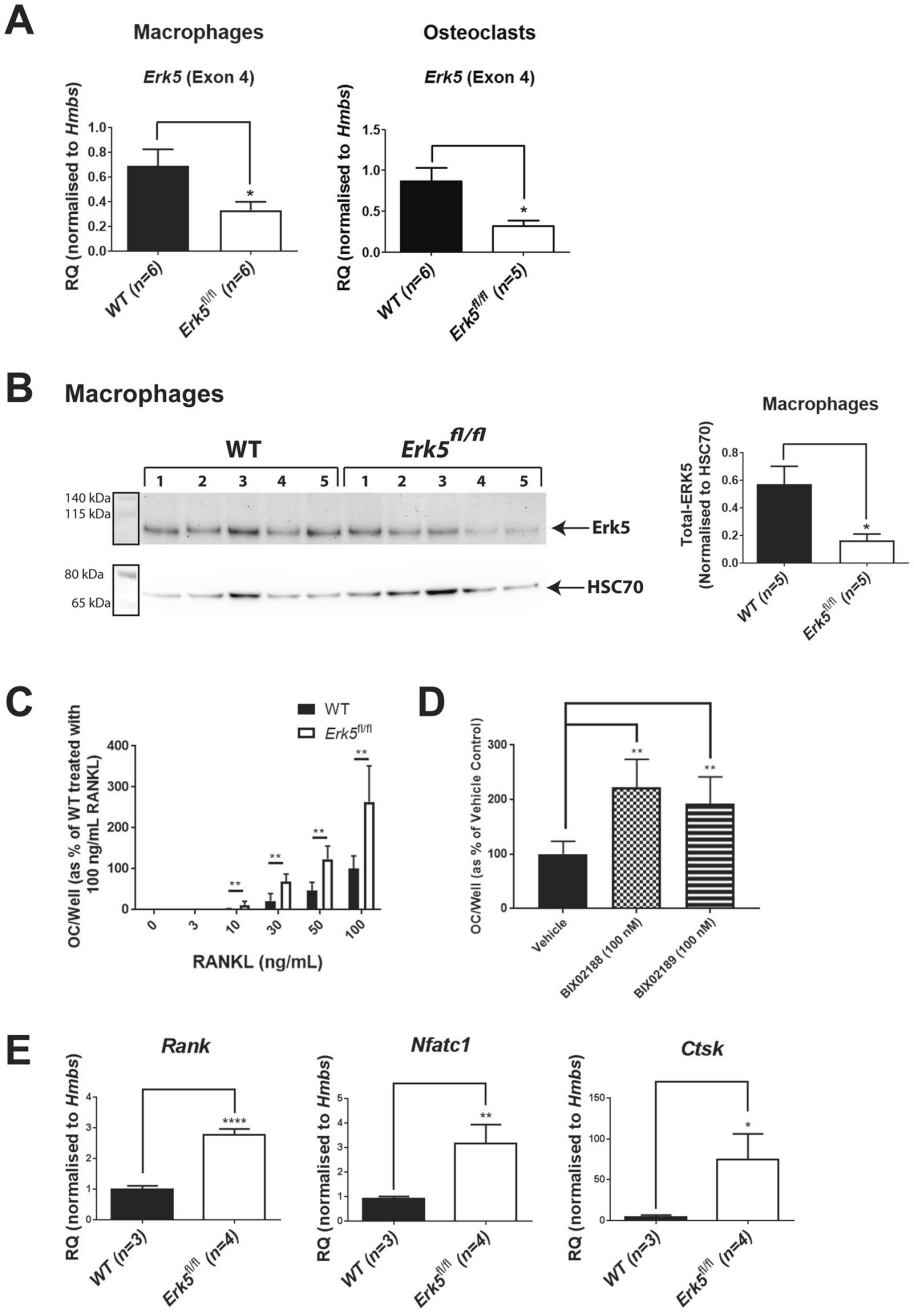
*Erk5* expression is significantly reduced in BMDM and mature osteoclast cultures from *Erk5* ^fl/fl^ mice compared to WT in parallel with a significant increase in osteoclast numbers and expression of the osteoclast markers, *Rank, Ctsk* and *Nfatc1*. (**A**) QPCR analysis of *Erk5* (Exon 4) mRNA expression (normalised to housekeeping gene, *Hmbs*) in BMDM and osteoclast cultures generated from *Erk5* ^fl/fl^ and WT mice. Shown are means; error bars represent SEM; t test (unpaired, 2 tailed) was used to calculate p values and those with significance are shown. *p < 0.05 from WT. (**B**) Western blot analysis (Left panel) of total ERK5 protein expression in BMDM cultures derived from WT (n = 5) and *Erk5* ^fl/fl^ (n = 5) mice. Black box highlights colorimetric image of molecular weight markers which was overlaid with chemiluminescent image of samples to confirm the size of observed bands. Full images are shown in Fig. S3. Densitometry analysis (right panel) was performed using image J to normalise total ERK5 levels to those of the housekeeping gene, HSC70. Shown are mean normalised expression levels of ERK5 for WT and *Erk5* ^fl/fl^; error bars represent SEM; t test (unpaired, 2 tailed) was used to calculate p values and those with significance are shown. *p < 0.05 from WT. (**C**) Osteoclast formation is increased in RANKL-stimulated BMDM cultures derived from *Erk5* ^fl/fl^ mice compared to WT. Figure shown is combined data from 3 independent experiments; n = 5 for each concentration of RANKL used within each experiment. Data was normalised to and is expressed as % of WT treated with 100 ng/mL RANKL. Shown are means; error bars represent SEM; ANOVA was used to calculate p values and those with significance are shown. **p < 0.01 from WT. **(D)** MEK5 inhibitors BIX02188 and BIX02189 (100 nM) stimulate osteoclast formation in WT cultures. Figure shown is combined data from 3 independent experiments; n = 5 for vehicle and each inhibitor used within each experiment. Data was normalised to and is expressed as % of vehicle control. Shown are means; error bars represent SEM; ANOVA was used to calculate p values and those with significance are shown. **p < 0.01 from vehicle. (**E**) QPCR analysis of *Rank, Ctsk* and *Nfatc1* mRNA expression (normalised to housekeeping gene, *Hmbs*) in BMDM and osteoclast cultures generated from *Erk5* ^fl/fl^ (n = 4) and WT (n = 3) mice. Shown are means; error bars represent SEM; t test (unpaired, 2 tailed) was used to calculate p values and those with significance are shown. *p < 0.05; **p < 0.05, ****p < 0.0001 from WT.

To further investigate the involvement of *Erk5* in the osteoclast compartment, macrophage colony-stimulating factor (M-CSF) dependent bone marrow-derived macrophages (BMDMs, osteoclast lineage pre-cursors) and osteoclasts (RANKL-stimulated BMDMs) were generated from the long bones of WT and *Erk5* ^fl/fl^ mice. Quantitative PCR (QPCR) was performed using RNA extracted from these BMDM and osteoclast cultures. BMDM and osteoclast cultures derived from *Erk5* ^fl/fl^ mice exhibited significantly lower expression of *Erk5* Exon 4 (the floxed region) when compared to WT controls (Fig. 4A and B), in keeping with an *Nkx3.1*:Cre mediated event. Moreover, BMDM cultures generated from *Erk5* ^fl/fl^ mice had significantly reduced levels of ERK5 protein compared to WT controls as assessed by Western blot (Fig. 4B and S3). To confirm reduced expression of *Erk5* in vertebral tissue sections, we tried immunohistochemical and *in situ* hybridization, by RNA Scope, staining of vertebral tissue sections for ERK5 protein and *Erk5* mRNA transcripts respectively but this was unsuccessful. To demonstrate successful areas of Cre recombination, a proportion of mice were bred to carry the *Z/EGFP* reporter transgene^27^. The *Z/EGFP* reporter mouse expresses lacZ throughout all embryonic and adult developmental stages. The expression of *Nkx3.1*:Cre during development leads to the excision of the *lacZ* gene, allowing expression of the second reporter, EGFP and so this essentially acts as a surrogate marker of Cre expression. GFP was found to be expressed in the macrophage and osteoclast cultures (Figure S2D) generated from *Nkx3.1*:Cre-expressing, *Z/EGFP* +ve mice, indicating the expression of Cre in the bone. RANKL dose-response experiments showed significantly increased numbers of osteoclasts in *Erk5* ^fl/fl^ cultures versus WT at concentrations of 10 ng/mL RANKL and above (Fig. 4C). Furthermore, the MEK5 inhibitors BIX02188 and BIX02189 stimulated RANKL-induced osteoclast formation in cultures from WT mice (Fig. 4D), suggesting that *Erk5* negatively regulates osteoclast differentiation. Next, we characterized the expression of a panel of osteoclast markers. Consistent with enhanced osteoclast differentiation, we found a significant increase in the expression of *Rank* (the receptor for RANKL), *Ctsk* (the gene encoding cathepsin K, the protease released by osteoclasts during bone resorption) and *Nfatc1* (a transcription factor that is crucial for osteoclast differentiation) in *Erk5* ^fl/fl^ cultures versus WT (Fig. 4E). Collectively, these data demonstrate that reduction of *Erk5* expression in the osteoclast compartment correlates with increased osteoclastogenesis and function.

## Discussion

While investigating the role of *Erk5* in the prostate using *Nkx3.1*-*Cre* recombinase expression, we observed a novel, dramatic spinal deformity in *Erk5* ^fl/fl^ mice. *Erk5* ^fl/fl^ mice were phenotypically normal at birth but developed the spinal defect by 2 months of age, suggesting that the loss of *Erk5* during the process of post-natal skeletal maturation is associated with the observed spinal phenotype. The spinal deformity was found to be linked with a profound reduction of trabecular bone mass in the vertebral column of both male and female *Erk5* ^fl/fl^ mice. From histomorphometry studies, we found that the low bone mass in male *Erk5* ^fl/fl^ mice was associated with increased bone-remodeling, with both significantly enhanced bone resorption and bone formation parameters being observed. It was striking that almost all (~90%) of the entire bone surface was undergoing re-modelling, either resorption or bone formation, in male *Erk5* ^fl/fl^ mice.

Low bone mass (osteopenia/osteoporosis) occurs when bone turnover is imbalanced in favour of bone resorption i.e. where the rate of bone resorption by osteoclasts exceeds the rate of bone formation by osteoblasts. Our dynamic histomorphometry data in male *Erk5* ^fl/fl^ mice point towards dis-regulated osteoclast function upon loss of *Erk5* as being the primary cause for the observed bone loss. However, we did not observe increased osteoclast surface or number in *Erk5* ^fl/fl^ female mice and the loss of bone mass was greater in male compared to female *Erk5* ^fl/fl^ mice, indicating that there are sex-specific differences. Gonadal sex steroid hormones (androgens and estrogens) are known to play an important role in bone homeostasis^28,29^. In particular, estrogen is known to inhibit osteoclastogenesis and osteoblast apoptosis^30^. Osteoporosis is a common disease in post-menopausal women, when estrogen levels have declined. It is established that while there is a net loss of bone mass, both bone resorption and bone formation rates are elevated in post-menopausal osteoporosis^31^, which is in keeping with our observations in male *Erk5* ^fl/fl^ mice. The spinal deformity develops in *Erk5* ^fl/fl^ female mice during the first two months of life and we suggest that the presence of estrogen in the female *Erk5* ^fl/fl^ mice at the time when bone histomorphometry parameters were assessed (10 weeks) mediates a protective effect in terms of inhibiting osteoclastogenesis/bone resorption and reducing the elevated bone formation rate when compared to male *Erk5* ^fl/fl^ mice. Testosterone levels in male *Erk5* ^fl/fl^ mice were found to be normal (data not shown) but future studies, beyond the scope of this work, to determine whether estrogen treatment could have a protective effect in the male *Erk5* ^fl/fl^ mice would be interesting and informative.

Development and maintenance of bone tissue involves precise spatial and temporal interplay between osteoclast and osteoblast function. RANKL is a key regulator of osteoclastogenesis and is negatively regulated by OPG. It is increasingly apparent that the major sources of both of these factors are osteoblasts and osteocytes, highlighting an important interplay between bone formation and bone resorption^32,33^. Our gross observations of the phenotype and extent of spinal deformity in *Erk5* ^fl/fl^ mice are similar to those from a classical study of *osteoprotegrin* (*OPG*)-deficient mice^34^, where it was reported that while embryonic bone formation appeared normal, adolescent and adult *OPG*-deficient mice exhibited a marked decrease in total bone density and trabecular bone porosity. Furthermore, the *OPG*-deficient mice also presented with curvature of the spine, had an increased incidence of vertebral bone fractures in the first two months of age and exhibited very active bone re-modeling, with increases in both osteoclast and osteoblast surface per bone surface. We examined the serum levels of RANKL and OPG in 7–11 week old WT and *Erk5* ^fl/fl^ male mice by ELISA and found that at this age, there was a significant reduction in the ratio of RANKL to OPG in *Erk5* ^fl/fl^ mice compared to WT, which would normally result in lower bone resorption levels. Although the RANKL/OPG ratio is very important for the maintenance of bone homeostasis, our results indicate that changes in this ratio are not what drives the low bone mass phenotype in these mice. Rather, this phenotype is most likely driven by increased responsiveness of the osteoclast precursors to RANKL, as suggested from our primary BMDM culture studies using a RANKL dose-response curve. The resulting increase in osteoclast formation could lead to bone loss *in vivo*, and this bone loss may result in the activation of compensatory mechanisms, such as the increased production of OPG and decreased RANKL production. Increased serum OPG levels have been observed in cases of post-menopausal osteoporosis^35^.

It is of interest that while *Erk5* has not been implicated in genome-wide association studies (GWAS) in the osteoporosis field to date^36^, a network-based meta analysis of gene expression profiles in women with bone mineral variations identified *Mekk3* (*Mek kinase 3*), which encodes an upstream kinase of MEK5 in the ERK5 pathway, as one of five candidate genes to be associated with bone mineral density^37^. Furthermore, *Mekk3* was identified as being induced by M-CSF in an oligonucleotide microarray study of gene expression patterns in mouse bone marrow mononuclear cells during the process of osteoclast differentiation^38^. Another upstream kinase of MEK5 in the ERK5 pathway, *Mekk2*, has previously been shown to be important in the regulation of osteoblast activity^39^. Collectively, our data are in keeping with a role for ERK5 pathway in normal bone homeostasis and clinically relevant osteoporosis.

Besides the spine, μCT of the long bones in the hind limbs also revealed significant loss of bone mass and trabecular structure in the *Erk5* ^fl/fl^ mice, indicating that there are effects in the appendicular skeleton as a result of *Nkx3.1*-mediated Cre recombinase expression. Our finding that *Erk5* expression is reduced in BMDM and osteoclast cultures support the notion of an impact of loss of *Erk5* in the appendicular skeleton. Examination of the growth plate from µCT images of distal femurs revealed that there was no major defect in growth plate cartilage/endochondral ossification, in male *Erk5* ^fl/fl^ mice compared to WT. However, the mineralised cartilage area appeared to be thinner, most probably due to the increased osteoclast activity in these mice. The additional phenotype of curly tail in a proportion of *Erk5* ^fl/fl^ mice is intriguing, and may result from the spine defect, similar to those seen in mouse models for spina bifida, neural tube defects and other transgenic mouse models^40,41^.

Importantly, we demonstrated that GFP, a surrogate marker for *Nkx3.1*:Cre activity, was expressed in BMDM and osteoclast cultures generated from *Nkx3.1*:Cre-expressing, *Z/EGFP* + ve mice. In parallel, *Erk5* mRNA expression was significantly reduced in BMDM and osteoclast cultures and total ERK5 protein expression was significantly reduced in BMDM cultures generated from *Erk5* ^fl/fl^ mice compared to WT, which is in keeping with Cre-mediated recombination having occurred. The efficiency of Cre-mediated recombination is never 100% and so our observed partial reduction in *Erk5* expression is indicative that deletion is occurring in only a subset of the cells. Despite this, osteoclast numbers and expression of osteoclast differentiation markers including *Rank*, *Ctsk* and *Nfatc1* were significantly increased in BMDM and osteoclast cultures generated from *Erk5* ^fl/fl^ mice compared to WT. As further validation of our findings in *Erk5* ^fl/fl^ cultures, we also demonstrated that inhibition of ERK5 pathway via treatment with the MEK5 inhibitors, BIX02188 and BIX02189, significantly increased osteoclast numbers in cultures generated from WT mice. Collectively, these data indicate that reduction of *Erk5* expression or activity in osteoclast pre-cursors is associated with increased osteoclastogenesis.

While our data indicate the osteoclast as being the primary compartment responsible for the observed bone loss, it is possible that other cell types could be affected by loss of *Erk5* expression driven by *Nkx3.1*-Cre. Interestingly, a recent study demonstrated that ERK5 is implicated in degenerated human intervertebral disc NP tissues^42^. ERK5 expression was found to be reduced in degenerated compared to normal tissues and treatment with TNF-α, which is implicated in NP cell degeneration, reduced *Erk5* mRNA expression and the expression of NP cell marker genes. In our study, the NP tissue between collapsed thoracic vertebrae in *Erk5* ^fl/fl^ mice was found to have an abnormal, distorted appearance whereas NP tissue located between thoracic vertebrae that had not collapsed in *Erk5* ^fl/fl^ mice appeared similar to WT. Furthermore, our histomorphometry studies were performed in lumbar vertebrae that showed no major deformation in intervertebral NP tissue, yet these vertebrae still show increased bone resorption. We suggest, therefore, that the NP deformation most likely results from vertebral collapse. Future studies to target *Erk5* ablation using an osteoclast-specific Cre, such as *Ctsk*-Cre, or other cell lineage-specific-Cre would be very informative to further address the question of the causal relationship between loss of *Erk5* in the osteoclast compartment with the spinal deformity.

To date, *in vitro* investigations of ERK5 in osteoblast and osteoclast biology have produced conflicting findings with both differentiation promoting and suppressive effects being documented^43–46^. Cyclic fluid shear stress has been shown to stimulate the proliferation of osteoblasts via an ERK5 signalling pathway^43,44^. However, in contrast to those studies, it has also been reported that the expression of key phenotypic markers for osteoblast differentiation, osteocalcin, alkaline phosphatase and osterix, are reduced after treatment with the MEK5 inhibitor, BIX02189, suggesting that MEK5 can suppress osteoblast differentiation^45^. Our data are in keeping with the former studies as we observe increased bone formation parameters in male *Erk5* ^fl/fl^ mice although we suggest that this could be an indirect effect of enhanced osteoclast activity. A very recent study has shown that ERK5 activation, through the induction of c-Fos, is essential for osteoclast-like differentiation of the monocytic RAW264.7D clone and 4B12 cells in response to RANKL and RANKL + M-CSF respectively^46^. The findings of Amano *et al*. are in contrast to our report as we demonstrate that reduction of *Erk5* expression, in the case of *Erk5* ^fl/fl^ mice, or inhibition of ERK5 pathway, in the case of treatment with small molecule MEK5 inhibitors, associated with increased osteoclast numbers and expression of osteoclast differentiation markers. Besides using primary bone marrow cells from our *in vivo* mouse model as opposed to cell lines, the ERK5 pathway inhibitor concentrations we used were considerably lower than those used by Amano *et al*. (100 nM versus 1–8 µM) because at high concentration of these inhibitors, we observed a high degree of toxicity and it is likely that selectivity for MEK5 is lost given that the reported IC_50_ for MEK5 kinase inhibition is 1.5 nM for both BIX0218 and BIX02189. Furthermore, Amano *et al*. used the inhibitor, XMD8-92, which is now known to have off-target effects against bromo domain-containing proteins^47^. For the first time, our study sheds important insights into the functional role of ERK5 in bone biology in an *in vivo* model.

In conclusion, we demonstrate an important role of *Erk5* in bone development and homeostasis *in vivo*. Loss of *Erk5* driven by *Nkx3.1*-Cre associated with a severe spinal deformity and an osteopenic phenotype. Furthermore, our evidence indicates that reduction of ERK5 pathway activity and *Erk5* gene expression promotes osteoclastogenesis. Our model could be of benefit to further our knowledge and understanding of the molecular events regulating bone homeostasis, spinal deformities and/or osteopenia.

## Materials and Methods

### Mouse Strains and Breeding

All small animal (murine) experiments were (i) approved by the Animal Welfare and Ethical Review Board (AWERB) at the University of Glasgow and (ii) performed in accordance with relevant guidelines and regulations. *Nkx3.1*:Cre mice^48^ were intercrossed with mice harbouring the conditional inactivatable *Erk5* allele (where Exon 4 is flanked by LoxP sites)^49^ to generate *Nkx3.1*:Cre;*Erk5* ^fl/+^ and *Nkx3.1*:Cre;*Erk5* ^fl/fl^ mice. A proportion of these mice were interbred with mice containing the *Z/EGFP* reporter transgene^27^. This transgene results in the expression of β-galactosidase in most tissues. The β-geo insert of the transgene is flanked by lox p sites and the expression of Cre recombinase results in the excision of the β-geo insert, activating the constitutive expression of GFP where Cre is expressed. Mice were genotyped by PCR by Transnetyx^TM^. Mice were of a mixed background (C57BL/6J) with strain-matched littermates (either Cre^+ve^ WT or mice not expressing Cre) used as control mice.

### Radiography

Whole body radiographs and radiographs of isolated spines of transgenic animals after sacrifice were performed using the Siemens Angiographie (AX) system. Exposure factors were: 40 kV; 50 mAs; 115 cm film to focus distance (FFD). Agfa CR 35-X developing cassettes were used.

### Histological Analysis

For histological analysis, mice were sacrificed using CO_2_ narcosis in order to maintain an intact skeleton, then bone tissues and a spectrum of other tissues, including liver, lung, kidney, heart, pancreas, prostate, leg muscle and small intestine, were collected and fixed intact in 10% formalin for 24 hr and subsequently processed for paraffin embedding. Sections from these paraffin blocks were stained with haematoxylin and eosin (H + E).

### μCT analysis

Whole mouse spines and 2-day-old pups were imaged using a Skyscan1076 *in vivo* μCT scanner (Skyscan, Kontich, Belgium) and were scanned at a resolution of 18 μm, with a source voltage of 50 kV, a 0.6° rotation step and a 0.5 mm aluminium filter. The scans were reconstructed using NRecon software, and visualised using CTVox and Dataviewer software (all Skyscan).

High resolution scans of mouse lumbar vertebrae and distal femurs were obtained using a Skyscan 1272 system at 4.5 μm resolution, 50 kV source voltage, 0.5° rotation step and a 0.5 mm aluminium filter. Scans were visualised as described above, and analysed using CtAn software (Skyscan). For the 5th lumbar vertebrae, the bone parameters were measured in the vertebral body, excluding the cortex and the end plates. For the distal femur, the analysis was performed in 200 slices 0.1 mm proximal of the primary spongiosa.

### Bone Histomorphometry

The mice received two injections of calcein (200 µl IP injection of 2 mg/ml) at 5 and 2 days before culling. Specimens were then fixed in 10% buffered formalin for 24 hr, and stored in 70% ethanol. After uCT analysis, the samples were dehydrated through an alcohol series, cleared in xylene, embedded in methyl methacrylate, and 5 μm sections were cut on a Leica RM2265 microtome.

For analysis of bone resorption parameters, the specimens were stained for TRAP to identify osteoclasts and the bone counterstained using Aniline Blue as described by Chappard *et al*.^50^. The sections were imaged on a Zeiss Axioimager microscope with a 10x lens and a QImaging Retiga 4000 camera resulting in a pixel size of 1.487 μm.

For analysis of bone formation parameters, the sections were stained without deplastification for 3 min in 0.1% Calcein Blue (Sigma), pH 8. The sections were washed twice in water, dehydrated through an alcohol series, cleared in xylene and coverslipped using Eukit (Sigma). The mineralised bone was imaged using a DAPI filter set, and the calcein labels using an FITC filter set on a Zeiss Axioimager microscope with a 20x lens and a QImaging Retiga 4000 camera resulting in a pixel size of 0.372 μm.

Bone histomorphometric analysis was performed using a custom in-house developed image analysis program based on ImageJ, available at https://www.liverpool.ac.uk/ageing-and-chronic-disease/bone-hist/ ^51^.

### RNA Isolation and Quantitation

RNA was isolated using RNeasy Mini Kit (Qiagen) as per manufacturer’s instructions, including a DNAse digestion step. RNA samples were quantified either by Qbit Assay (Invitrogen) according to manufacturer’s instructions or using a NanoDrop™ spectrophotometer (Thermo Scientific).

### Quantitative Real Time Polymerase Chain Reaction (QPCR) – Taqman

cDNA was prepared from RNA samples using High Capacity cDNA Transcription Kit (Applied Biosystems). QPCR was performed to evaluate relative transcript expression levels in cDNA samples using the Taqman technique. Specific primer/probe combinations (Table S6) and Taqman Universal PCR Mastermix (Applied Biosystems) were added to cDNA samples (according to manufacturer’s instructions) before being run on an Applied Biosystems 7500 Fast Real-Time PCR System machine. The QPCR conditions were: 20 sec at 50 °C, 10 min at 95 °C, followed by 40 cycles of 15 sec at 95 °C and 1 min at 60 °C. Technical replicates for each sample and a minimum of three independent samples for each genotype/cell line were included*. Hmbs*, the gene encoding hydroxymethylbilane synthase, was used as the ‘house-keeping’ or reference gene and 7500 Software v2.0.5 (Applied Biosystems) used 2^−ΔΔCT^ method to determine the relative gene expression. Primer/Probe sets were designed using the Universal Probe Library (UPL) Assay design centre (Roche).

### Enzyme-linked immunosorbent assay (ELISA)

Serum samples were collected from mouse whole blood by centrifugation for 15 min at approximately 800 × *g*. We used the RANKL (TNFSF11) Mouse ELISA Kit (ab100749) and Osteoprotegerin Mouse ELISA Kit (ab100733) (ELISA) from Abcam® (Cambridge, UK) to measure the serum levels of RANKL and OPG in 7–11 week old male WT and *Erk5* ^fl/fl^ mice, according to manufacturer’s instructions. Serum samples were diluted 1:15 for RANKL ELISA and 1:50 for OPG ELISA using Diluent A which was provided with the kits. Standard curves and experimental samples were performed in duplicates. Data are expressed as nanograms per milliliter.

### Generation and culture of BMDMs and osteoclasts

Bone marrow cells were flushed out of the long bones of the mice and the cell suspension cultured in αMEM supplemented with 10% FCS, Pen/Strep and 100 ng/ml M-CSF (Prospec Bio) for three days. Next the non-adherent cells were removed by washing the cultures with PBS, and the adherent BMDMs harvested using Acutase (Sigma) or cell dissociation buffer (Enzyme-Free; PBS-based) (Gibco, Life Technologies). For osteoclast formation experiments, cells were plated in 96-well plates at 15 × 10^3^ cells in 100 µl medium supplemented with 25 ng/ml M-CSF and up to 100 ng/ml RANKL (a kind gift of Dr. J. Dunford, University of Oxford) per well. The culture medium was refreshed at day 2 and day 4 and the cells fixed on day 5 with 4% buffered formalin and stained for TRAP. TRAP positive cells containing three or more nuclei were counted as osteoclasts. For RNA isolation, BMDM cells were seeded in 6-well plates at 5 × 10^5^ cells per well in medium supplemented with 25 ng/ml M-CSF only (for macrophage cultures) or 25 ng/ml M-CSF plus 100 ng/ml RANKL (for osteoclast cultures) and cultured for 5 days as described above. For Western Blot analysis, BMDM cells were seeded in 6-well plates at 5 × 10^5^ cells per well in medium supplemented with 25 ng/ml M-CSF and cultured for 3 days.

### Western Blotting

Whole cell lysates (WCL) were prepared by lysing cells in lysis buffer [50 mM Tris pH 7.6, 150 mM NaCl, 1% Triton X-100, 0.5% deoxycholate, 0.1% SDS, 1 mM sodium ortho-vanadate, 5 mM sodium fluoride, 50 µg/mL phenylmethylsulfonyl fluoride (PMSF), protease inhibitor cocktail mix 1 (Calbiochem) and PhosSTOP (Roche)]. WCL were resolved by SDS-PAGE on 4–12% gradient polyacrylamide gels (Invitrogen) at 180 V for 1 hr and transferred electrophoretically by wet transfer system onto PVDF membrane (Milipore) at 100 V for 1 hr. Blots were blocked for 1 hr with 5% skimmed milk, rinsed and probed with the required primary antibody (diluted in 5% BSA, 0.1% Tween-20 containing TBS and 0.05% sodium azide) overnight at 4 °C. Following incubation with appropriate horseradish peroxidase-conjugated secondary antibody (Cell Signalling, 7076 or 7074, 1:5000) (in 5% skimmed milk), bands were visualised using Bio-RAD ChemiDoc™ Imaging System and software. Antibodies used were: ERK5 (Cell Signalling, 3372, 1:1000) and HSC70 (B-6) (Santa Cruz Biotechnology, sc-7298, 1:1000). Densitometry was performed using Image J software.

### Statistical analysis

Statistical analyses (t-test; ANOVA, using Dunnett’s post-hoc test) were performed using GraphPad Prism v7.02 or SPSS v21 software. For all graphs, mean ± SEM (error bars) are presented.

## Electronic supplementary material

Supplementary Information
