# Supplementary material for: Analysis of Nkx3.1:Cre-driven Erk5 deletion reveals a profound spinal deformity which is linked to increased osteoclast activity
Source: Sci Rep. 2017 Oct 16;7:13241. doi: 10.1038/s41598-017-13346-8 (PMC5643304; doi:10.1038/s41598-017-13346-8)
Supplement: Supplementary file 1 — Supplementary Information [file 41598_2017_13346_MOESM1_ESM.pdf]

Supplementary File: Analysis of *Nkx3.1*:Cre-driven *Erk5* deletion reveals a profound spinal deformity which is linked to increased osteoclast activity.

Authors: Carolyn J. Loveridge[1,2], Rob J. van 't Hof[3,†], Gemma Charlesworth[3], Ayala King[1,2], Ee Hong Tan[2], Lorraine Rose[4], Anna Daroszewska[3], Amanda Prior[3], Imran Ahmad[1,2], Michelle Welsh[5], Ernest J. Mui[2], Catriona Ford[2], Mark Salji[1,2], Owen Sansom[2], Karen Blyth[2], and Hing Y. Leung[1,2,*]

Author affiliations

[1] Institute of Cancer Sciences, College of Medical, Veterinary and Life Sciences, University of Glasgow, Bearsden, Glasgow, G61 1BD, UK.

[2] Beatson Institute for Cancer Research, Bearsden, Glasgow, G61 1BD, UK.

[3] Institute of Ageing and Chronic Disease, University of Liverpool, WH Duncan Building, West Derby Street, Liverpool, L7 8TX, UK.

[4] Centre for Molecular Medicine, MRC IGMM, University of Edinburgh, Edinburgh EH4 2XU, UK.

[5] College of Medical, Veterinary and Life Sciences, University of Glasgow, Glasgow G61 1QH, UK.

***To whom correspondence should be addressed**

Prof. Hing Y. Leung, Beatson Institute for Cancer Research, Bearsden, Glasgow, G61 1BD, UK, e-mail: h.leung@beatson.gla.ac.uk, tel: 44(0) 141 330 3658, fax: 44(0) 141 942 6521

[†] Alternative corresponding author

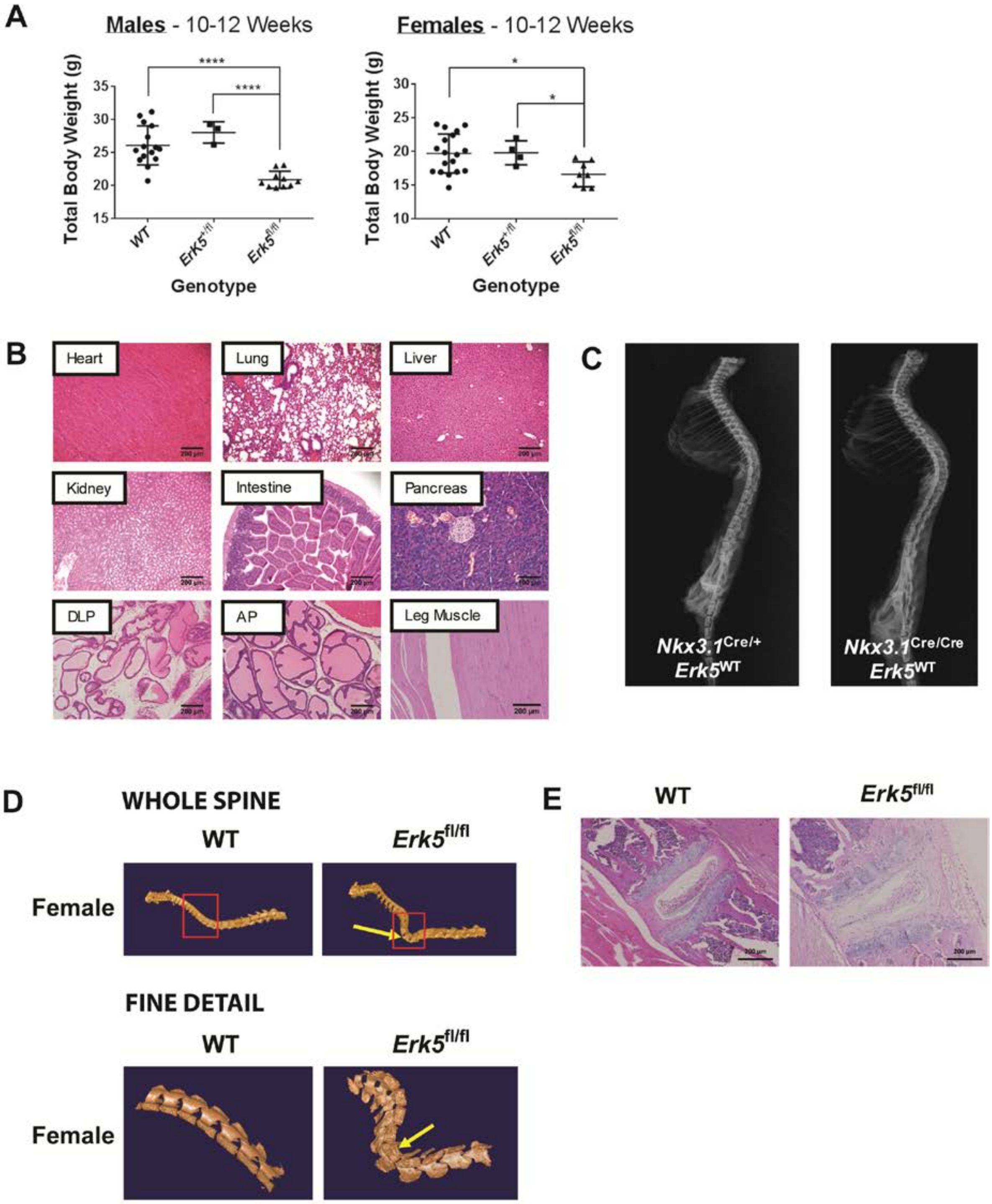

A
Males - 10-12 Weeks
Females - 10-12 Weeks
Total Body Weight (g)
****
*
WT
Erk5+/fl
Erk5fl/fl
Genotype
B
Heart
Lung
Liver
Kidney
Intestine
Pancreas
DLP
AP
Leg Muscle
C
Nkx3.1Cre/+ Erk5WT
Nkx3.1Cre/Cre Erk5WT
D
WHOLE SPINE
WT
Erk5fl/fl
Female
FINE DETAIL
E

Figure S1

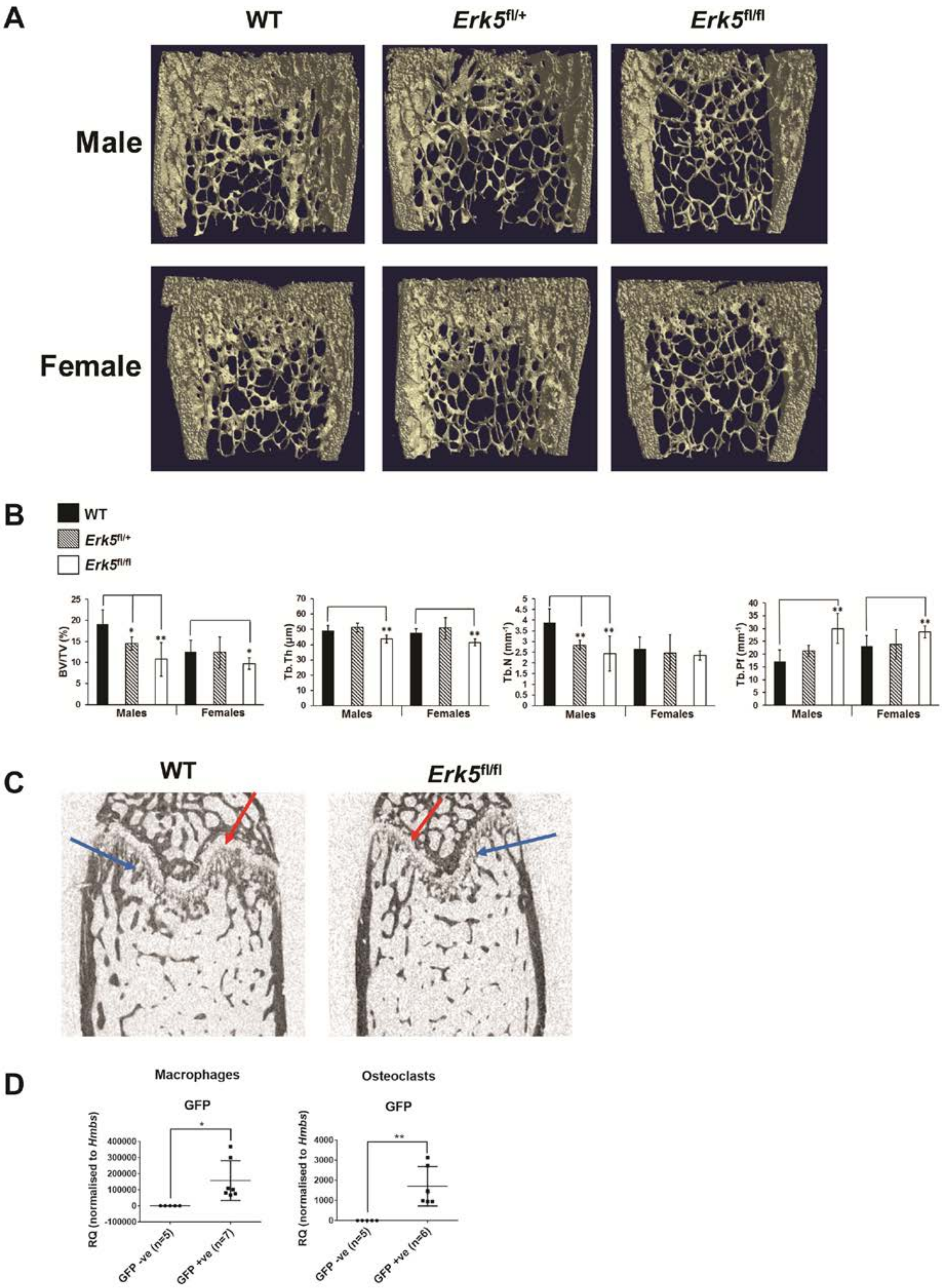
A
WT
Erk5fl/+
Erk5fl/fl
Male
Female
B
WT
Erk5fl/+
Erk5fl/fl
BV/TV (%)
Tb.Th (µm)
Tb.N (mm-1)
Tb.Pf (mm-1)
Males
Females
C
WT
Erk5fl/fl
D
Macrophages
Osteoclasts
GFP
RQ (normalised to Hmbs)
GFP -ve (n=5)
GFP +ve (n=7)
GFP +ve (n=6)

Figure S2

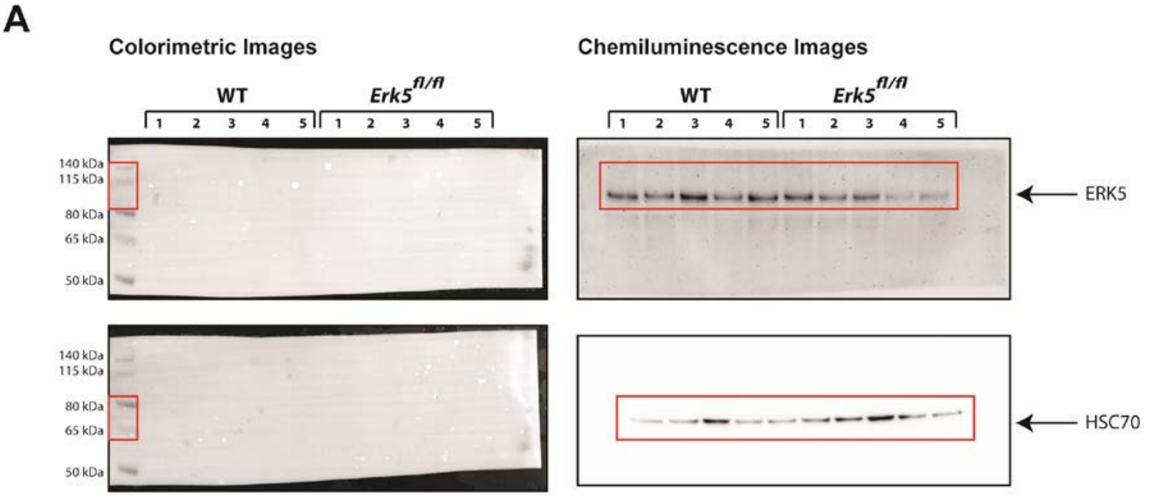
A
Colorimetric Images
Chemiluminescence Images
WT
Erk5 fl/fl
1 2 3 4 5
140 kDa
115 kDa
80 kDa
65 kDa
50 kDa
ERK5
HSC70

Figure S3

Legends to Supplementary Figures

<u>Figure S1</u> - *Erk5*$^{fl/fl}$ mice were significantly smaller than *Erk5*$^{fl/+}$ and WT littermates with no abnormalities detected in other major organs or prostate in *Erk5*$^{fl/fl}$ mice. No spinal phenotype was observed in *Nkx3.1*$^{Cre/Cre}$ mice. Female *Erk5*$^{fl/fl}$ mice have severe spinal deformities in the thoracic region but intervertebral disc nucleus pulposus tissue between thoracic vertebrae that had not collapsed in female *Erk5*$^{fl/fl}$ mice appeared normal.

(**A**) Male and female *Erk5*$^{fl/fl}$ mice at 10-12 weeks of age weighed significantly less compared to WT and *Erk5*$^{fl/+}$ mice. Shown in (**A**) are means; error bars represent SEM; t test (unpaired, 2 tailed) was used to calculate p values and those with significance ($p<0.05$) for comparisons made are specified.; *: $p<0.05$; ****: $p<0.0001$ from same sex WT. (**B**) Representative images of H+E staining of heart, lung, liver, kidney, intestine, pancreas, dorsal lateral prostate (DLP), anterior prostate (AP) and leg muscle in *Erk5*$^{fl/fl}$ mice. (**C**) Representative X-ray images of isolated spines from 12 week old *Nkx3.1*$^{Cre/+}$;*Erk5*^{WT} and *Nkx3.1*$^{Cre/Cre}$;*Erk5*^{WT} mice. (**D**) Representative images of low resolution µCT of isolated spines from female *Erk5*$^{fl/fl}$ mice. In the bottom panel, fine detail images of female spines are shown. Yellow arrows in top panel denote deformation of the mutant spinal column. Note the wedge-shaped deformation of the mutant vertebral body highlighted by yellow arrow in the fine detail view of mutant vertebrae. Red boxes in the top panel indicate the regions of the spines shown in finer detail in the bottom panel. (**E**) Representative micrographs (10x) of H+E staining of longitudinal sections from vertebrae of 5 month old female WT and *Erk5*$^{fl/fl}$ mice. The thoracic vertebrae shown for *Erk5*$^{fl/fl}$ are not collapsed and there is no major malformation of intervertebral disc nucleus pulposus tissue between these vertebrae; the histology is very similar to WT.

Figure S2 - Distal femurs of *Erk5*$^{fl/fl}$ mice have reduced trabecular number, thickness and reduced bone volume but there was no major defect in endochondral ossification. *GFP* expression is detected in bone marrow-derived macrophage (BMDM) and osteoclast cultures in *Nkx3.1*:Cre-expressing, *Z/EGFP* +ve mice.

(**A**) Representative images of high resolution µCT of distal femur in 11 week old male and female WT and *Erk5*$^{fl/fl}$ mice. (**B**) Quantitative analysis of high resolution µCT parameters indicate a significant reduction in trabecular BV/TV (%) and Tb.Th (µm) in male and female *Erk5*$^{fl/fl}$ mice compared to WT and a significant increase in Tb.Pf (mm^{-1}) in male and female *Erk5*$^{fl/fl}$ mice compared to WT. Tb.N (mm^{-1}) is reduced in male *Erk5*$^{fl/+}$ and *Erk5*$^{fl/fl}$ mice compared to WT. Abbreviations: BV/TV: bone volume per tissue volume; Tb.Th: trabecular thickness; Tb.SP: trabecular separation; Tb.N: trabecular number; Tb.Pf: trabecular pattern factor. Shown in the graphs are means; error bars represent SEM; t test (unpaired, 2 tailed) was used to calculate p values and those with significance ($p<0.05$) for comparisons made are specified; *: $p<0.05$; **: $p<0.01$ from same sex WT. (**C**) Representative images of µCT (4.5 µm) of growth plate in distal femurs of 10-12 week old male WT and *Erk5*$^{fl/fl}$ mice. Red arrows point to the growth plate cartilage; blue arrows point to the mineralised cartilage. (**D**) QPCR analysis of *GFP* mRNA expression (normalised to housekeeping gene, *Hmbs*) in BMDM and osteoclast cultures generated from *Nkx3.1*:Cre-expressing *Erk5*$^{fl/fl}$ and WT mice, which were segregated on the basis of *Z/EGFP* status (either positive or negative). Shown in both panels are means; error bars represent SEM; t test (unpaired, 2 tailed) was used to calculate p values and those with significance ($p<0.05$) for comparisons made are specified; *: $p<0.05$; **: $p<0.01$.

<u>Figure S3</u> - Full colorimetric and chemiluminescent images relating to Figure 4B.

(**A**) Shown are the full length and colorimetric images which were used to prepare Figure 4B. The molecular weight ladder in the colorimetric images was overlaid with the chemiluminescent images to aid identification of the molecular weight of observed bands. Highlighted by red boxes are areas of the images which were cropped

Supplementary Tables

<u>Table S1</u> – Genotype numbers, penetrance of spinal phenotype and hind limb/curled tail phenotype and total body weight at 10-12 weeks (Males)

**Genotype**	**Age Range (Weeks)**	**Number**	**Spinal Phenotype**	**Curled Tail**	**Total Body Weight (g) (at age 10-12 weeks)**
**WT**	10-18	18	0	0	26.06 ± 0.7623 (n=15)
Erk5 $^{\mathbf{fl/+}}$	7-18	7	0	0	28.01±0.9286 (n=3)
Erk5 $^{\mathbf{fl/fl}}$	17-18	17	17	5	20.86 ± 0.3997 (n=10) ****

<u>Statistical Information</u>: t test (unpaired, 2 tailed) was used to calculate p values and those with significance ($p<0.05$) are specified; ****: $p<0.0001$ from same sex wild type and same sex heterozygotes

Table S2 – Genotype numbers, penetrance of spinal phenotype and hind limb/curled tail phenotype and total body weight at 10-12 weeks (Females)

**Genotype**	**Age Range (Weeks)**	**Number**	**Spinal Phenotype**	**Curled Tail**	**Total Body Weight (g) (at age 10-12 weeks)**
**WT**	10-28	25	0	0	19.68 ± 0.6833 (n=18)
Erk5$^{fl/+}$	7-26	10	0	0	19.79 ± 0.8872 (n=4)
Erk5$^{fl/fl}$	7-37	16	16	5	16.59 ± 0.6529 (n=8) *

Statistical Information: t test (unpaired, 2 tailed) was used to calculate p values and those with significance ($p<0.05$) are specified; *: $p<0.02$ from same sex wild type and same sex heterozygote

Table S3 – Analysis of trabecular bone structure of the 5th lumbar vertebra by µCT

	**Male**			**Female**		
	WT	***Erk5*** $^{fl/+}$	***Erk5*** $^{fl/fl}$	***WT***	***Erk5*** $^{fl/+}$	***Erk5*** $^{fl/fl}$
**BV/TV (%)**	22.23±2.82	18.81±1.69*	10.20±2.65**	21.04±2.28	20.79±2.70	13.68±2.59**
**Tb.Th (µm)**	43.28±3.84	43.52±2.79	43.19±2.21	43.39±2.48	46.13±4.36	46.06±2.83
**Tb.Sp (µm)**	173.8±17.1	199.3±14.5*	270.7±34.9**	198.0±13.4	213.2±22.1	259.1±11.1**
**Tb.N (mm^{-1})**	5.13±0.41	4.32±0.31**	2.36±0.56**	4.86±0.52	4.51±0.44	2.96±0.49**
**Tb.Pf (mm^{-1})**	6.37±0.27	9.09±0.95*	21.30±4.49**	5.81±2.44	6.31±3.39	14.58±2.85**
**Conn.Dn (mm^{-3})**	578.2±170.6	412.5±57.4	260.3±71.2**	495.9±126.5	450.5±90.3	343.1±90.9*
**SMI**	0.82±0.18	0.99±0.05	1.72±0.22**	0.82±0.13	0.86±0.22	1.44±0.29**

Abbreviations: BV/TV: bone volume per tissue volume; Tb.Th: trabecular thickness; Tb.SP: trabecular separation; Tb.N: trabecular number; Tb.Pf: trabecular pattern factor; Conn.Dn: connectivity density; SMI: structure model index.

Genotype Numbers: Males – WT (n=12), *Erk5* $^{fl/+}$ (n=6), *Erk5* $^{fl/fl}$ (n=6); Females – WT (n=9), *Erk5* $^{fl/+}$ (n=7), *Erk5* $^{fl/fl}$ (n=6)

Statistical Information: t test (unpaired, 2 tailed) was used to calculate p values and those with significance ($p<0.05$) are specified; *: $p<0.05$; **: $p<0.01$ from same sex wild type.

<u>Table S4</u> – Analysis of trabecular bone structure of the distal femur by μCT

	**Male**			**Female**		
	WT	***Erk5**$^{fl/+}$*	***Erk5**$^{fl/fl}$*	***WT***	***Erk5**$^{fl/+}$*	***Erk5**$^{fl/fl}$*
**BV/TV (%)**	19.10±3.45	14.54±1.55*	10.75±3.97**	12.58±2.76	12.40±3.70	9.74±1.35*
**Tb.Th (μm)**	49.31±3.31	51.16±2.87	43.70±2.59**	47.60±2.74	50.82±6.89	41.50±2.37**
**Tb.Sp (μm)**	168.5±17.7	199.4±11.8**	209.7±38.6**	217.69±23.2	224.6±36.0	215.2±12.7
**Tb.N (mm^{-1})**	3.87±0.66	2.84±0.22**	2.44±0.82**	2.65±0.56	2.47±0.84	2.35±0.22
**Tb.Pf (mm^{-1})**	17.1±4.5	21.3±2.1	30.0±5.8**	23.1±4.1	23.8±5.8	28.7±2.3**
**Conn.Dn (mm^{-3})**	412.1±138.2	290.3±43.7	326.6±122.6	312.5±96.7	269.0±111.5	306.7±52.0

<u>Abbreviations</u>: BV/TV: bone volume per tissue volume; Tb.Th: trabecular thickness; Tb.SP: trabecular separation; Tb.N: trabecular number; Tb.Pf: trabecular pattern factor; Conn.Dn: connectivity density; SMI: structure model index.

<u>Genotype Numbers</u>: Males – WT (n=12), *Erk5*$^{fl/+}$ (n=6), *Erk5*$^{fl/fl}$ (n=6); Females – WT (n=9), *Erk5*$^{fl/+}$ (n=7), *Erk5*$^{fl/fl}$ (n=6)

<u>Statistical Information</u>: t test (unpaired, 2 tailed) was used to calculate p values and those with significance ($p<0.05$) are specified; *: $p<0.05$; **: $p<0.01$ from same sex wild type.

<u>Table S5</u> – Dynamic histomorphometry of the 5th lumbar vertebra

	**Male**			**Female**		
	WT	***Erk5$^{fl/+}$***	***Erk5$^{fl/fl}$***	***WT***	***Erk5$^{fl/+}$***	***Erk5$^{fl/fl}$***
**BV/TV (%)**	30.13±3.13	24.00±5.71*	15.04±4.76**	27.90±2.79	24.68±5.56	19.39±2.90**
**Oc.S/BS (%)**	12.75±5.34	13.99±5.79	21.53±2.75**	33.38±7.35	33.85±5.36	29.47±8.10
**N.Oc/BS (mm^{-1})**	5.02±1.62	7.50±2.85*	7.35±0.98**	10.82±2.72	10.97±1.39	10.19±2.36
**MAR (ìm/day)**	2.16±0.68	2.75±0.55	3.42±0.13	2.99±0.19	3.02±0.32	2.97±0.41
**MS/BS (%)**	32.44±8.12	39.83±14.66	56.10±2.37***	51.58±5.89	48.81±2.82	49.08±6.458
**BFR/BS (mm^{-3})**	0.72±0.33	1.16±0.66	1.93±0.15**	1.55±0.26	1.48±0.22	1.65±0.16

<u>Abbreviations</u>: BV/TV: bone volume per tissue volume; Oc.S/BS: osteoclast surface per bone surface; N.Oc/BS: number of osteoclasts per bone surface; MAR: mineral aposition rate; MS/BS: mineralising surface per bone surface; BFR/BS: bone formation rate per bone surface.

<u>Genotype numbers for bone volume and osteoclast parameter analysis</u> (TRAP): Males – WT (n=12), *Erk5*$^{fl/+}$ (n=6), *Erk5*$^{fl/fl}$ (n=6); Females – WT (n=9), *Erk5*$^{fl/+}$ (n=7), *Erk5*$^{fl/fl}$ (n=6).

<u>Genotype numbers for calcein double label analysis</u>: Males – WT (n=9), *Erk5*$^{fl/+}$ (n=4), *Erk5*$^{fl/fl}$ (n=6); Females – WT (n=9), *Erk5*$^{fl/+}$ (n=4), *Erk5*$^{fl/fl}$ (n=3).

Statistical Information: t test (unpaired, 2 tailed) was used to calculate p values and those with significance ($p<0.05$) are specified; *: $p<0.05$; **: $p<0.01$; ***: $p<0.001$ from same sex wild type.

<u>Table S6</u> – Primer pair sequences and UPL probe used for each gene studied

Gene	Forward Primer	Reverse Primer	UPL Probe
Erk5 **(Exon 4)**	5'-TTACCAGGGAGCGCATTAAG-3'	5'-GTCGTGCATGGAAGTCCTC-3'	5
Hmbs	5'-TCCCTGAAGGATGTGCCTAC-3'	5'-AAGGGTTTTCCCGTTTGC-3'	79
Rank (Tnfrs11a)	5'-GTGCTGCTCGTT CCACTG-3'	5'-AGATGCTCATAATGCCTCTCCT-3'	25
Cathepsin K	5'-CGAAAAGAGCCTAGCGAACA-3'	5'-TGGGTAGCAGCAGAAACTTG-3'	18
Nfatc1	5'-TCCAAAGTCATTTTCGTGGA-3'	5'-TTTGCTTCCATCTCCCAGAC-3'	50
GFP	5'-GAAGCGCGATCACATGGT-3'	5'-CCATGCCGAGAGTGATCC-3'	67
